# Evaluation of femoral head bone quality by Hounsfield units: A predictor of implant failure for intertrochanteric fractures after intramedullary nail fixation

**DOI:** 10.3389/fsurg.2022.816742

**Published:** 2023-01-06

**Authors:** Jixing Fan, Yang Lv, Xiangyu Xu, Fang Zhou, Zhishan Zhang, Yun Tian, Hongquan Ji, Yan Guo, Zhongwei Yang, Guojin Hou

**Affiliations:** ^1^ Department of Orthopedics, Peking University Third Hospital, Beijing, China; ^2^ Engineering Research Center of Bone and Joint Precision Medicine, Ministry of Education, Beijing, China

**Keywords:** intertrochanteric fracture, Hounsfield units, bone quality, implant failure, intramedullary nail

## Abstract

**Purpose:**

The aim of present study is to evaluate the femoral head bone quality by Hounsfield units and its relationship to the occurrence of implant failure for intertrochanteric fractures after intramedullary nail fixation.

**Methods:**

This retrospective study assessed 160 intertrochanteric fractures treated with intramedullary fixation. Patients with and without implant failure were divided into failure and control groups, respectively. The demographic information, femoral head Hounsfield unit (HU) value, the reduction quality, status of posteromedial support and position of the screw/blade were collected and compared. The logistic regression analyses were performed to evaluate risk factors of implant failure in intertrochanteric fractures after intramedullary nail fixation.

**Results:**

Of the patients, 15 (9.38%) suffered from implant failure after intramedullary fixation. The mean HU value of femoral head was much lower in the failure group than the control group (133.25 ± 34.10 vs. 166.12 ± 42.68, *p* = 0.004). And the univariate analyses showed that A3 fracture and poor reduction quality were associated with implant failure (*p* < 0.05). After adjustment for confounding variables, the multivariable logistic regression analyzes showed that femoral head HU value (odds ratio [OR], 0.972; 95% CI, 0.952–0.993; *p* = 0.008) and poor reduction quality (OR, 7.614; 95% CI, 1.390–41.717; *p* = 0.019) were independent influencing factors for implant failure.

**Conclusion:**

The femoral head HU value was significantly correlated with the incidence of implant failure and can be used as an independent factor to predict implant failure for intertrochanteric fractures after intramedullary fixation.

## Introduction

Intertrochanteric fractures are very common in the elderly patients, which have substantial mortality, morbidity, and healthcare costs. The major reason is osteoporosis, which is characterized by low bone mass and destruction of the bone microarchitecture (1). With the increase of the aging population, osteoporosis has gradually become a global health problem that affects nearly 200 million people worldwide (2). Furthermore, older patients are reported to have a higher rate of osteoporosis than the general population (3). Consequently, the incidence of intertrochanteric fractures is increasing quickly over the past several decades owing to the absolute increase in the elderly population, and this trend is expected to continue (4).

Intramedullary nail fixation is a common procedure used in the surgical treatment of intertrochanteric fractures (5, 6). Implant failure is one of the main reasons for secondary surgical interventions after initial surgical treatment of intertrochanteric fractures. It has been investigated that intramedullary nails have a failure rate ranging from 3% to 22% (7–9). Although several risk factors have been investigated to be associated with the occurrence of implant failure, poor bone quality is one of the influencing factors which could increase the risk of early mechanical failure (10, 11). However, there is a relative scarcity of literature describing the relation between low bone mineral density (BMD) and implant failure in intertrochanteric fractures after intramedullary nail fixation.

BMD measured in Hounsfield units (HU) on computed tomography (CT) is used in the evaluation of BMD and can serve as a diagnostic tool for osteoporosis (12). Although it has been reported that there is a significant correlation between HU values and dual energy x-ray absorptiometry (DXA) measurements, few studies have been carried out on the application of femoral head HU values to the prediction of implant failure for intertrochanteric fractures after intramedullary nail fixation. In the present study, we retrospectively reviewed patients who received surgical intervention with intramedullary nail fixation for intertrochanteric fractures, and we investigated possible correlations between femoral head HU values and the incidence of implant failure in these patients.

## Materials and methods

### Source of patients

This retrospective study was performed at a level 1 trauma center over a 6-year period from January 1, 2012, to December 31, 2018. Patients who were surgically treated by the same trauma surgeon group in our department for intertrochanteric fractures were retrospectively included in current study. Included patients should meet the following criteria: (1) age 60 years or older; (2) acute intertrochanteric fracture (<2 weeks from injury); (3) low-energy injury, a low-energy injury was defined as an injury which patients would sustain while falling over slippery ground in a walking or sitting position; (4) underwent CT scans of the injured hip before surgery; (5) intramedullary nail (IMN) fixation; (6) a minimum follow-up period of 12 months or until the time of implant failure. Exclusion criteria were as follows: (1) pathological fracture (secondary to tumor or primary hyperparathyroidism); (2) delayed fracture; (3) stress fracture; (4) open fracture, or ipsilateral knee or ankle fractures; (5) periprosthetic fracture; (6) previous history of hip surgery; (7) presence of other circumstances that affect bone metabolism, such as long-term steroid use; and (8) femoral heads with any pathologies such as focal lytic or sclerotic lesions. The demographic information (including age, gender, side of injury, and body mass index [BMI]) were collected.

### Measurement of femoral head HU value

**Figure 1:**
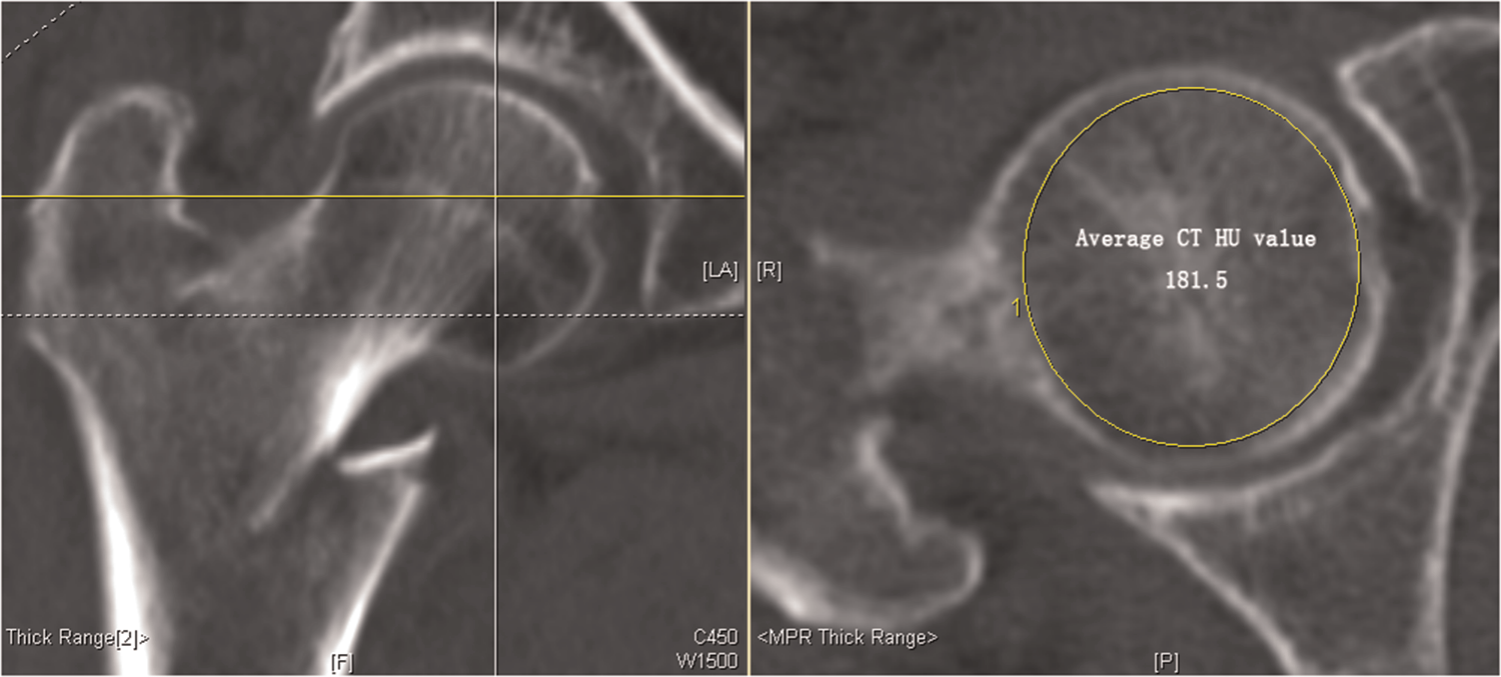
The methods of measuring computed tomography (CT) Hounsfield units (HU) values of the femoral head. A single axial femoral head level was selected. The level included the last slice of femoral neck–first slice of femoral head combination. A spherical region of interest (ROI) was drawn with the largest possible diameter excluding the cortical bone in femoral head and average HU value (181.5 HU) was noted. CT, computed tomography; ROI, region of interest.

A preoperative hip CT scan (Defnition, Siemens) was performed for all of the included patients. The tube voltage of all of the CT scans was set at 120 kV. An experienced trauma surgeon (JF) independently used a PACS system (GE Electrics, Fairfield, USA) to measure the femoral head HU value. In order to facilitate measurement and repetition, we selected a single axial femoral head level. The combination of the first slice of femoral head and the last slice of femoral neck was selected. A spherical region of interest (ROI) was drawn with the largest possible diameter excluding the cortical bone (Figure 1). The reasons for choosing this region are as followed: (I) this area is the main area through which tensile trabeculae and compressive trabeculae pass together, which are weakened in osteoporosis; (II) the spherical ROI can be easily measured; and (III) it is a well-known location to ensure that measurements are performed from the same point (13).

### Surgical technique

All operations were performed by five experienced orthopedic doctors. The type of anesthesia was either spinal anesthesia or general anesthesia. All patients were treated with the reduction and internal fixation. According to the manufacturer's protocol, intramedullary devices were implanted in routine surgical procedure. The number, model, and trade mark of the IM devices were summarized in Table 1. The patient received antibiotics and deep venous thrombosis prevention postoperatively. When the x-ray showed the appearance of fracture healing, partial weight-bearing was initiated, and total weight-bearing began after clinical fracture healing. The follow-up evaluation was performed at 1, 3, 6, 12 months after the surgery and yearly thereafter.

**Table 1:** Utilization of implant types.

Implant type	Failure rate
Gamma 3 (Stryker, Mahwah, NJ)	2/34 (5.88%)
Proximal femoral nail antirotation (PFNA, Synthes USA, Paoli, PA)	13/105 (12.38%)
TRIGEN InterTan nail (Smith & Nephew, Inc., Memphis, USA)	0/17
Proximal femoral nail (PFN, Synthes USA, Paoli, PA)	0/1
TRIGEN Tan nail (Smith & Nephew, Inc., Memphis, USA)	0/3

### Outcome assessment

The reduction quality, status of posteromedial support and position of the screw/blade were used to evaluate the outcome by postoperative radiographs immediately after surgery.

The reduction quality was described as good, acceptable, or poor, according to the modified criteria of Baumgaertner et al. (14) and and Kim et al. (15), which was addressed by measuring alignment and displacement of main fragments on the AP and lateral view.

According to the extent of displacement of the posteromedial fragment, the status of the posteromedial support was defined as existence or loss. Good alignment and a displacement of less than the cortical thickness were defined as existence. Otherwise, the status of the lateral femoral wall was recorded as loss (16).

The tip-apex distance (TAD) was used to access the position of the screw/blade in the femoral head. The TAD was determined by measuring the distance from the tip of the helical blade to the apex of the femoral head on both anteroposterior (AP) and lateral radiographs (14). The amount of radiographic magnification was determined precisely by the known diameter of the helical blade.

**Figure 2:**
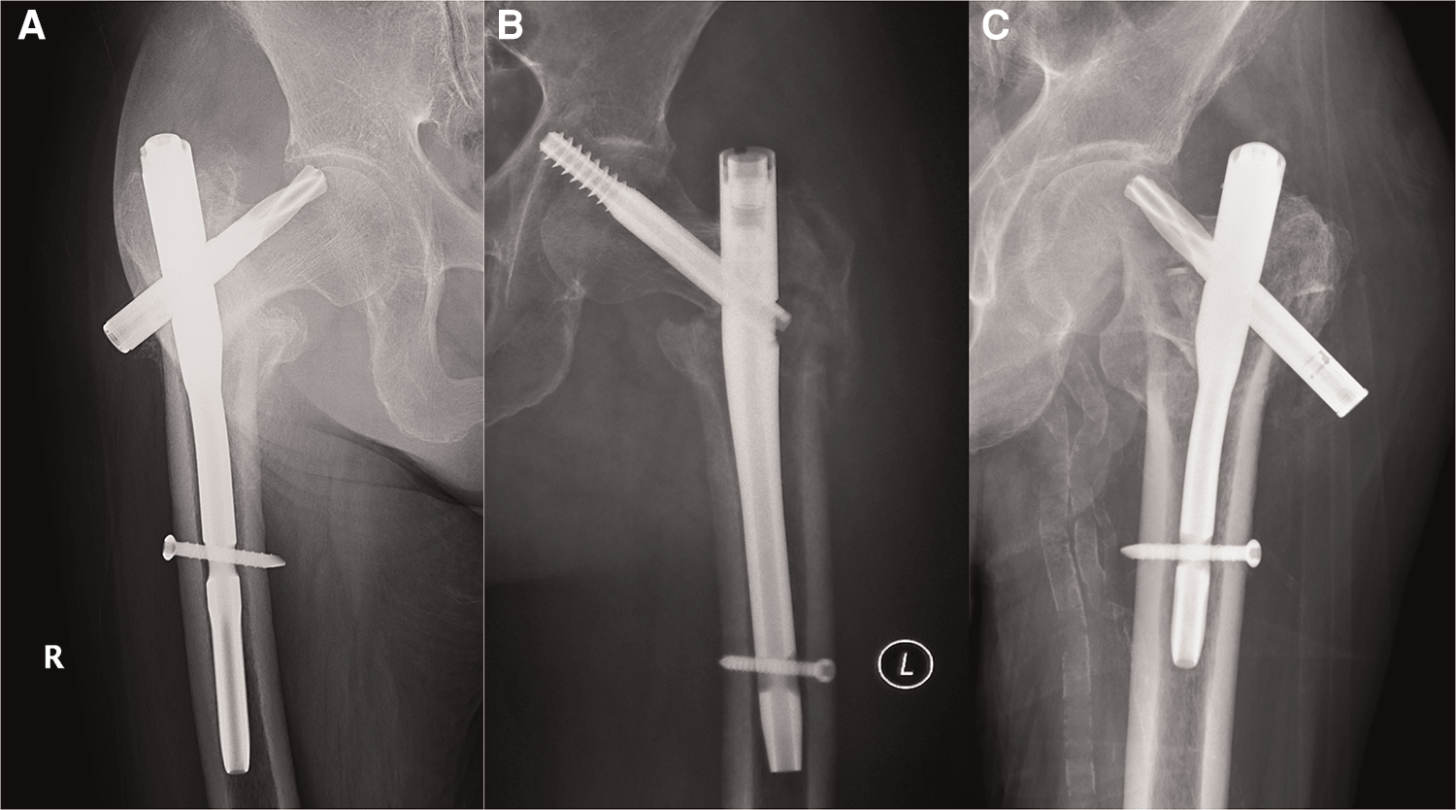
Types of implant failure. (**A**) Screw/blade cut out; (**B**) screw/blade perforation; (**C**) screw/blade back out.

In this study, helical blade cut out, perforation and back out were defined as the implant failure. The perforation of the helical blade through the superior cortex of the femoral head or neck was defined as the blade cut out. The penetration of the helical blade from the femoral head into the surrounding soft tissues and hip joint was defined as the blade perforation (Figure 2).

### Statistical analysis

In this study, the SPSS 21.0 software was used for statistical analysis (SPSS, Chicago, Illinois, USA). For quantitative data, the normal distribution was evaluated by the one-sample Kolmogorov–Smirnov test. Continuous variables were compared by the independent sample Student t-test or the Mann–Whitney test as appropriate. For qualitative data, the *χ*^2^ test was used. Risk factors of implant failure in intertrochanteric fractures after intramedullary nail fixation were evaluated by the multivariate logistic regression analysis when the factor's *p* value was <0.1. The likelihood ratio backward test was conducted to find the best-fit model by selecting variables one by one. A *p* value <0.05 was considered statistically significant, and all tests were two sided.

## Results

In the present study, a total of 160 patients were included in the final analysis. The patient's age ranged from 60 years to 97 years, and the mean age was 77.37 (±8.81) years. Among these patients, 15 patients (9.38%) suffered from implant failure. The mean failure time was 5.7 months (ranging from 1 to 27 months). Of these 15 patients with implant failure, cut out occurred in six patients, blade perforation occurred in two patients, and blade back out occurred in seven patients.

In the present study, patients were divided into control group and failure group according to patients with and without implant failure. The comparison of general characteristics between the control group and failure group was shown in Table 2. The results showed that there were significant differences between control group and failure group in AO classification, femoral head HU value and reduction quality (*p* < 0.05). Specially, the mean HU value of femoral head was much lower in the failure group than the control group (133.25 ± 34.10 vs. 166.12 ± 42.68, *p* = 0.004). However, the age, gender, injury side, BMI, TAD, posteromedial support, and NSA showed no significant differences between control group and failure group (*p* > 0.05).

**Table 2:** The comparison of patient characteristics between control group and failure group.

Variable	Control group (*n* = 145)	Failure group (*n* = 15)	OR (CI 95%)	*p* Value
Age (years)	77.15 ± 8.76	79.47 ± 9.30	—	0.334
Gender				
Male	49 (33.8%)	4 (26.7%)	1.0 (reference)	—
Female	96 (66.2%)	11 (73.3%)	1.404 (0.425–4.637)	0.578
Side of injury				
Left	80 (55.2%)	8 (53.3%)	1.0 (reference)	—
Right	65 (44.8%)	7 (46.7%)	1.077 (0.371–3.127)	0.892
BMI (kg/cm^2^)	23.41 ± 3.84	24.31 ± 3.15	—	0.385
AO classification				
A1	35 (24.1%)	2 (13.3%)	1.0 (reference)	—
A2	96 (66.2%)	8 (53.3%)	1.458 (0.295–7.202)	0.643
A3	14 (9.7%)	5 (33.3%)	6.250 (1.083–36.076)	0.040*
Femoral head HU value	166.12 ± 42.68	133.25 ± 34.10	—	0.004*
TAD (mm)	19.80 ± 5.01	21.45 ± 5.51	—	0.231
Reduction quality				
Good	113 (77.9%)	8 (53.3%)	1.0 (reference)	—
Acceptable	27 (18.6%)	4 (26.7%)	2.093 (0.587–7.463)	0.255
Poor	5 (3.4%)	3 (20.0%)	8.475 (1.709–42.016)	0.009*
Posteromedial support				
Existence	100 (69.0%)	9 (60.0%)	1.0 (reference)	
Loss	45 (31.0%)	6 (40.0%)	0.675 (0.227–2.010)	0.480
NSA	130.24 ± 3.31	130.89 ± 3.29	—	0.471

BMI, body mass index; AO, Arbeitsgemeinschaft fur Osteosynthesefragen; HU, Hounsfield unit; TAD, tip apex distance; NSA, neck shaft angle; OR, odds ratio; CI, confidence interval.

* The difference was significant.

The results of the adjusted multivariate logistic regression analyses were presented in Table 3. After adjustment for other risk factors, the results showed that femoral head HU value (odds ratio [OR], 0.972; 95% CI, 0.952–0.993; *p* = 0.008) and poor reduction quality (OR, 7.614; 95% CI, 1.390–41.717; *p* = 0.019) were independent influencing factors for implant failure whereas the AO classification of intertrochanteric fracture was not associated with implant failure (*p* > 0.05).

**Table 3:** Multivariable logistic regression analysis for risk factors associated with implant failure.

Predictor	Adjusted OR	95% CI	*p* Value
Femoral head HU value	0.972	0.952–0.993	0.008*
AO classification			
A1	1.0 (reference)	—	—
A2	1.086	0.204–5.787	0.923
A3	3.873	0.571–26.256	0.165
Reduction quality			
Good	1.0 (reference)	—	—
Acceptable	1.602	0.425–6.042	0.487
Poor	7.614	1.390–41.717	0.019*

HU, Hounsfield unit; AO, Arbeitsgemeinschaft fur Osteosynthesefragen; OR, odds ratio; CI, confidence interval. The multivariable regression analysis used backward selection with use of the likelihood ratio test to assess significance.

* The difference was significant.

To identify better predictors for implant failure, the receiver operating curve (ROC) analysis was performed, and area under the curve (AUC) values were calculated for femoral head HU. The femoral head HU had a good AUC value (0.724). The cut-off value of femoral head HU was 124.85, with a sensitivity of 84.8% and specificity of 53.3%.

## Discussion

The DXA is the most commonly used parameter for the evaluation of BMD. However, the DXA had some shortcomings inherent to the imaging modality itself, such as an inability to both estimate true volumetric bone density as well as discriminate between cortical and trabecular bone mas (17, 18). The usage of CT, particularly quantitative computed tomography (QCT), has been investigated in evaluating the osteoporosis (19, 20). In addition, the QCT has been shown to be effective in distinguishing cortical bone from trabecular bone; however, this technique relies on the use of phantoms for calibration during acquisition (21). Furthermore, due to the high cost of equipment, the use of QCT software requires strict training. At present, it is difficult to apply this technology in many clinical environments (12). A HU is a dimensionless unit unique to CT examination. BMD evaluation based on HU value is correlated with the standard BMD value and physical strength of the bone, which has been widely reported (22). Furthermore, the femoral head HU obtained from routine CT could be used to evaluate regional bone quality of femoral head (13). Thus, theoretically, the CT HU value is an effective predictor of BMD-related complications after surgical treatment of intertrochanteric fractures. However, limited studies have investigated its use in assessing the femoral head bone quality in intertrochanteric fractures.

In current literature, the overall implant failure rate was 9.38%. Furthermore, we found that patients in the failure group had a significantly lower mean HU value of femoral head than those in the control group. Significant differences were also found in AO classification and reduction quality between control group and failure group. In addition, after adjustment for confounding variables, the multivariate logistic regression analysis showed that a lower femoral head HU value and poor reduction quality were independent influencing factors for implant failure whereas the AO classification of intertrochanteric fracture was not associated with the implant failure.

With regard to the stability of reconstructions after intertrochanteric fracture reduction and fixation, five influencing factors had been summarized by Kaufer et al. (5), that is, bone quality (osteoporosis), the choice of implant, the quality of reduction, the placement of the implant in femoral head, and the fragment geometry. Growing biomechanical evidence had suggested that osteoporosis was an important risk factor for mechanical failure in intertrochanteric fractures (23, 24). In this study, we found that the femoral head HU value was significantly lower in the failure group than that in the control group. Furthermore, multivariate logistic regression analysis showed that a lower femoral head HU value was an independent risk factor of implant failure. The ROC analysis showed that the cut-off value of femoral head HU was 124.85. This meant that the femoral head HU value below 124.85 was associated with increased risk of implant failure. With an AUC value of 0.724, the femoral head HU value was deemed as a reliable predictor of implant failure. The possible reason might be that poor bone quality could challenge the anchorage of the load carrier in the femoral head. Therefore, cement-augmented screws or arthroplasty (joint replacement) might be an alternative for the surgical treatment of intertrochanteric fractures with poor bone quality.

A previous study had developed a novel method based on voxel-based morphometry for estimating nail-tract bone density in intertrochanteric fracture patients treated with intramedullary nails by 3D image analysis software (25). However, the screw pathway's CT HU value could not be measured directly with routine PACS on axial images of the femoral head. If we only focus on the CT HU value of the screw's pathway, the measurement might be complex and could not be applied in routine clinical practice. Thus, we chose the routine method to measure the preoperative CT HU value of the femoral head to predict implant failure, which was convenient and simple.

It was well known that the fracture geometry had a significant impact on the outcome of surgical treatment for intertrochanteric fractures. Classification of intertrochanteric fracture served as a guideline for treatment and helped to predict the result or provided a reasonable estimation of the likely outcome (26). In some previous reports, relatively high complication rates were noted in patients with unstable intertrochanteric fractures (27). In the present study, patients with A3 fracture tended to have greater risk of implant failure compared with A1 fracture (OR, 6.250; 95% CI, 1.083–36.076; *p* = 0.040) in the univariate analyses. After adjusting confounding factors, we found that patients with the A3 fracture remained a nearly four-fold greater risk compared with A1 fracture in the multivariate analyses; however, no statistically significant difference was observed. This result was consistent with a previous study and implied that the fracture geometry could influence the stability of intertrochanteric fractures; this result was necessary to be verified in the future.

In the present study, for intertrochanteric fractures after intramedullary nail fixation, we demonstrated that a poor reduction quality was an independent risk factor for the occurrence of implant failure (OR, 7.614; 95% CI, 1.390–41.717; *p* = 0.019). This was consistent with previous studies, which revealed that fracture reduction quality was one of the paramount factors in maintaining the stability of the intertrochanteric fracture after surgical treatment (6, 28). The possible reason might be that cortical continuity could contribute to the ability of the cortex to resist collapse; whereas, Mal-angulation, in particular varus mal-angulation, might increase bone-implant stresses and risk of collapse (29, 30). The reduction quality had been described as good, acceptable, or poor according to the alignment and displacement of main fragments (14, 15). Previous studies had shown that good reduction quality could reduce the risk of mechanical failure in intertrochanteric fractures whereas poor reduction quality was associated with the occurrence of implant failure (7, 31). Therefore, great emphasis should be paid on the fracture reduction quality in the future.

The main advantage of this study was that it was the first study to evaluate the femoral head HU value on CT and its relationship to implant failure for intertrochanteric fractures after intramedullary nail fixation. Nevertheless, several limitations existed in this study. First, this was a retrospective study. Second, the sample size in this study was relatively small. Third, the ROIs for HU measurements were selected manually, which might raise concerns about the repeatability of these results. However, it had been proven that HU measurements had excellent interobserver and intraobserver reliability (32). Fourth, with the calibration of the device, the HU value could change. This was the fundamental limitation of the studies performed by HU. In further studies, it might be necessary to repeat the study using different devices and software.

## Conclusion

In conclusion, we found that the HU value was significantly correlated with the incidence of implant failure and can be used as an independent factor to predict implant failure for intertrochanteric fractures after intramedullary fixation. Therefore, CT scan was a convenient and simple tool to evaluate femoral head bone quality. In addition, poor reduction quality could increase the risk of implant failure for intertrochanteric fractures after intramedullary fixation. Thus, we should pay attention to the reduction quality in the future.

## Data Availability

The raw data supporting the conclusions of this article will be made available by the authors, without undue reservation.
